# Supplementary material for: Identification of limiting climatic and geographical variables for the distribution of the tortoise Chelonoidis chilensis (Testudinidae): a baseline for conservation actions
Source: PeerJ. 2015 Oct 1;3:e1298. doi: 10.7717/peerj.1298 (PMC4636419; doi:10.7717/peerj.1298)
Supplement: Supplemental Information 1 — Appendix S1: Bayesian spatially expanded logistic (BSEL) model and model selection procedure Table S1: Complete list of observations and sources. Table S2: Explanatory variables and model selection. Fig. S1: BSEL model uncertainty Table S3: Presence of Chelonoidis chilensis on protected areas in Argentina and Bolivia. [file peerj-03-1298-s001.docx]

**SUPPORTING INFORMATION**

**Identification of limiting climatic and geographical variables for the distribution of the tortoise *Chelonoidis chilensis* (Testudinidae)**

*Authors*: Alejandro Ruete^1*^, Gerardo C. Leynaud^2^

**Appendix S1. Bayesian spatially expanded logistic (BSEL) model and model selection procedure**

We developed a Bayesian spatially expanded logistic (BSEL) model (Casetti 1997; Congdon 2003) to obtain the probability of observation at non-visited locations. Non-visited locations were randomly located with the same density as the observed locations (~0.0004/km^2^). Given the nature of presence-only data, predicted probabilities combine the probability of the species being at the location, the probability of an observer being at the same location, and the probability of the observer finding the species (Lobo *et al.* 2010). We assume that observations at every non-visited location *i* are distributed according to a Bernoulli distribution *Obs_i_* ~ Bernoulli(*p^*^_i_*), where *p^*^_i_* is an *a priori* probability distribution generated from confirmed observations (Fig. 2b). We generated the *a priori* probability distribution as a quadratic density kernel raster layer using the R package “*splancs*” (Rowlingson *et al.* 2013). By generating a prior distribution from the observations, we assume that the entire study region has been sampled with the same intensity.

We then modelled observations *Obs_i_* according to a logistic model, *Obs_i_* ~ Bernoulli(*p_i_*), and logit(*p_i_*) = φ + α*_v_* · **V** + β*_v,i_* · **V**, where *p_i_* is the probability of observing the species at location *i*, logit the logistic link function, φ is the regression intercept, and **V**_v×i_ is a matrix for the environmental variables *v*. The spatially expanded model (Casetti 1997; Congdon 2003) assumes that the effect of an explanatory variable *v* on the response variable *p_i_* varies among the observed locations. This assumption is particularly convenient when fitting species distribution model along large ranges, where the species can be locally adapted to e.g. temperature ranges (Turchin & Hanski 1997; Nilsson-Örtman *et al.* 2013). The model allows estimating fixed parameters (α*_v_*; i.e. without spatial variability) and flexible parameters (β*_v,i_*) that correct for the spatial variation of the effect parameter α*_v_*, as well as for spatial autocorrelation on the observations. The parameter β*_v,i_* is further modelled as β*_v,i_* = *x_i_* · γ*_xv_* + *y_i_* · γ*_yv_*, where γ*_xv_* y γ*_yv_* are correction parameters for coordinates *x_i_* and *y_i_*. The combined effect (fixed and flexible) of variable *v* varies for every location, and is described as δ*_v,i_* = α*_v_* + β*_v,i_*, with an average of $\bar{\delta}_{v}=\alpha_{v}+\bar{\beta}_{v}$.

The final model presented (Table 1) is the result of a selection procedure based on the deviance information criterion (DIC), an information-theoretic criterion that is appropriate for Bayesian hierarchical modelling (Spiegelhalter et al. 2002). The lower the DIC, the better the model is able to predict a new data set, and thus, the DIC penalizes for increasing model complexity just as the commonly used Akaike’s information criterion (AIC; Burnham & Anderson 2002). First, we compared models for each independent environmental variable with a null model (i.e. only including the intercept parameter φ). Then, we built the final model with a forward stepwise procedure, following the DIC ranking of the variables (Tables S2). We used a threshold of 10 DIC units to consider model improvement. A model could include correlated variables if the later added variable improved the model fit, according to the over-parameterized models criterion (Reichert & Omlin 1997).

**References**

Casetti, E. (1997). The expansion method, mathematical modeling, and spatial econometrics. *International Regional Science Review*, **20**, 9–33. Retrieved August 18, 2012,

Congdon, P. (2003). *Applied Bayesian modelling*. John Wiley and Sons, West Sussex, England.

Lobo, J.M., Jiménez-Valverde, A. & Hortal, J. (2010). The uncertain nature of absences and their importance in species distribution modelling. *Ecography*, **33**, 103–114. Retrieved September 23, 2013,

Nilsson-Örtman, V., Stoks, R., De Block, M., Johansson, H. & Johansson, F. (2013). Latitudinally structured variation in the temperature dependence of damselfly growth rates. *Ecology Letters*, **16**, 64–71. Retrieved March 15, 2013,

Reichert, P. & Omlin, M. (1997). On the usefulness of overparameterized ecological models. *Ecological Modelling*, **95**, 289–299. Retrieved January 11, 2010,

Rowlingson, B., Diggle, P., Bivand, R., Petris, G. & Eglen, S. (2013). *splancs: Spatial and space-time point pattern analysis*. Retrieved from http://CRAN.R-project.org/package=splancs

Turchin, P. & Hanski, I. (1997). An empirically based model for latitudinal gradient in vole population dynamics. *The American Naturalist*, **149**, 842–874. Retrieved May 23, 2012,

**Table S1.** Complete list of observations

| ID | Source | X | Y |
| --- | --- | --- | --- |
| 1 | 1 | -62.883 | -38.483 |
| 2 | 4 | -60.283 | -22.350 |
| 3 | 1 | -62.618 | -39.810 |
| 4 | 2 | -64.250 | -27.733 |
| 5 | 4 | -64.760 | -40.600 |
| 6 | 4 | -62.453 | -23.467 |
| 7 | 4 | -65.541 | -41.042 |
| 8 | 1 | -66.669 | -37.155 |
| 9 | 9 | -67.615 | -37.353 |
| 10 | 9 | -64.564 | -37.285 |
| 11 | 1 | -62.816 | -41.030 |
| 12 | 3 | -60.000 | -22.500 |
| 13 | 3 | -62.980 | -40.800 |
| 14 | 3 | -62.680 | -39.480 |
| 15 | 3 | -65.470 | -28.050 |
| 16 | 3 | -61.200 | -27.220 |
| 17 | 4 | -60.450 | -26.780 |
| 18 | 4 | -60.430 | -26.350 |
| 19 | 3 | -61.170 | -26.520 |
| 20 | 9 | -60.620 | -25.950 |
| 21 | 3 | -61.280 | -27.320 |
| 22 | 3 | -60.220 | -26.870 |
| 23 | 3 | -64.230 | -31.480 |
| 24 | 3 | -63.970 | -31.620 |
| 25 | 4 | -63.930 | -31.650 |
| 26 | 3 | -64.570 | -31.480 |
| 27 | 2 | -63.430 | -30.750 |
| 28 | 4 | -63.620 | -31.330 |
| 29 | 3 | -63.580 | -30.350 |
| 30 | 3 | -63.580 | -30.400 |
| 31 | 3 | -58.680 | -32.470 |
| 32 | 3 | -67.500 | -30.000 |
| 33 | 3 | -63.580 | -30.150 |
| 34 | 3 | -67.680 | -37.800 |
| 35 | 3 | -67.620 | -37.370 |
| 36 | 3 | -66.930 | -36.250 |
| 37 | 3 | -66.230 | -37.550 |
| 38 | 3 | -65.920 | -38.150 |
| 39 | 3 | -66.400 | -38.720 |
| 40 | 3 | -65.650 | -37.320 |
| 41 | 3 | -64.600 | -37.380 |
| 42 | 3 | -66.820 | -28.550 |
| 43 | 3 | -68.000 | -33.470 |
| 44 | 3 | -68.070 | -33.580 |
| 45 | 3 | -68.000 | -33.850 |
| 46 | 3 | -67.970 | -34.050 |
| 47 | 3 | -68.400 | -34.580 |
| 48 | 3 | -67.900 | -34.830 |
| 49 | 3 | -67.900 | -34.830 |
| 50 | 3 | -68.250 | -34.830 |
| 51 | 3 | -67.920 | -34.220 |
| 52 | 3 | -67.880 | -34.480 |
| 53 | 3 | -67.900 | -34.600 |
| 54 | 3 | -68.780 | -32.970 |
| 55 | 3 | -68.100 | -38.030 |
| 56 | 3 | -65.680 | -39.270 |
| 57 | 3 | -65.480 | -39.100 |
| 58 | 3 | -64.430 | -40.100 |
| 59 | 3 | -65.250 | -40.400 |
| 60 | 3 | -65.250 | -40.400 |
| 61 | 3 | -63.970 | -24.870 |
| 62 | 3 | -67.330 | -31.480 |
| 63 | 3 | -67.780 | -32.200 |
| 64 | 3 | -65.370 | -32.770 |
| 65 | 3 | -62.830 | -28.470 |
| 66 | 2 | -63.000 | -28.470 |
| 67 | 2 | -63.470 | -29.370 |
| 68 | 3 | -64.500 | -27.400 |
| 69 | 3 | -63.450 | -29.550 |
| 70 | 3 | -63.700 | -29.520 |
| 71 | 3 | -63.950 | -29.050 |
| 72 | 3 | -64.900 | -27.520 |
| 73 | 3 | -62.850 | -26.580 |
| 74 | 3 | -64.830 | -28.000 |
| 75 | 3 | -65.280 | -26.220 |
| 76 | 3 | -57.920 | -22.330 |
| 77 | 1 | -62.580 | -39.770 |
| 78 | 3 | -64.250 | -27.730 |
| 79 | 3 | -64.480 | -30.200 |
| 80 | 9 | -60.620 | -25.980 |
| 81 | 2 | -64.270 | -27.780 |
| 82 | 3 | -58.680 | -32.470 |
| 83 | 3 | -64.970 | -40.750 |
| 84 | 3 | -64.080 | -38.970 |
| 85 | 4 | -63.000 | -40.750 |
| 86 | 3 | -64.630 | -37.420 |
| 87 | 3 | -68.080 | -38.920 |
| 88 | 3 | -64.230 | -27.730 |
| 89 | 3 | -64.080 | -39.100 |
| 90 | 3 | -68.770 | -33.000 |
| 91 | 3 | -64.180 | -31.400 |
| 92 | 3 | -58.500 | -34.670 |
| 93 | 3 | -68.400 | -34.580 |
| 94 | 3 | -65.500 | -40.500 |
| 95 | 3 | -60.670 | -32.950 |
| 96 | 4 | -59.300 | -23.100 |
| 97 | 4 | -57.880 | -22.600 |
| 98 | 3 | -60.000 | -22.500 |
| 99 | 4 | -65.006 | -40.616 |
| 100 | 5 | -65.373 | -41.565 |
| 101 | 1 | -62.249 | -40.555 |
| 102 | 1 | -67.085 | -39.623 |
| 103 | 1 | -64.692 | -40.088 |
| 104 | 8 | -62.646 | -40.184 |
| 105 | 8 | -63.006 | -39.587 |
| 106 | 8 | -63.677 | -38.879 |
| 107 | 8 | -65.169 | -39.227 |
| 108 | 8 | -65.405 | -39.500 |
| 109 | 8 | -64.535 | -40.171 |
| 110 | 8 | -65.367 | -43.291 |
| 111 | 8 | -64.883 | -42.322 |
| 112 | 8 | -65.430 | -41.688 |
| 113 | 8 | -65.367 | -40.992 |
| 114 | 8 | -63.777 | -40.880 |
| 115 | 8 | -62.820 | -40.743 |
| 116 | 8 | -64.684 | -40.631 |
| 117 | 8 | -64.932 | -40.457 |
| 118 | 8 | -65.206 | -40.333 |
| 119 | 8 | -65.492 | -40.495 |
| 120 | 8 | -65.827 | -40.606 |
| 121 | 8 | -66.623 | -39.786 |
| 122 | 8 | -66.536 | -39.277 |
| 123 | 8 | -67.170 | -39.214 |
| 124 | 8 | -66.349 | -38.605 |
| 125 | 8 | -65.840 | -38.146 |
| 126 | 8 | -65.541 | -38.046 |
| 127 | 8 | -65.343 | -37.425 |
| 128 | 8 | -64.423 | -37.400 |
| 129 | 8 | -66.026 | -37.412 |
| 130 | 8 | -67.766 | -37.885 |
| 131 | 8 | -68.239 | -37.822 |
| 132 | 8 | -67.393 | -37.773 |
| 133 | 8 | -67.530 | -37.251 |
| 134 | 8 | -66.660 | -36.343 |
| 135 | 8 | -68.189 | -36.244 |
| 136 | 8 | -67.431 | -35.772 |
| 137 | 8 | -67.915 | -35.809 |
| 138 | 8 | -67.741 | -35.548 |
| 139 | 8 | -67.990 | -35.424 |
| 140 | 8 | -68.276 | -35.299 |
| 141 | 8 | -68.089 | -35.063 |
| 142 | 8 | -67.766 | -34.902 |
| 143 | 8 | -68.189 | -34.765 |
| 144 | 8 | -68.015 | -37.524 |
| 145 | 8 | -67.688 | -34.380 |
| 146 | 8 | -67.436 | -34.068 |
| 147 | 8 | -67.791 | -34.157 |
| 148 | 8 | -67.643 | -33.950 |
| 149 | 8 | -67.836 | -33.757 |
| 150 | 8 | -68.192 | -33.638 |
| 151 | 8 | -68.563 | -33.401 |
| 152 | 8 | -67.851 | -33.505 |
| 153 | 8 | -67.658 | -33.253 |
| 154 | 8 | -67.050 | -33.460 |
| 155 | 8 | -66.575 | -33.223 |
| 156 | 8 | -67.169 | -33.104 |
| 157 | 8 | -67.539 | -32.912 |
| 158 | 8 | -67.228 | -32.778 |
| 159 | 8 | -66.739 | -33.015 |
| 160 | 8 | -67.361 | -32.585 |
| 161 | 8 | -67.495 | -32.422 |
| 162 | 8 | -67.791 | -32.467 |
| 163 | 8 | -67.895 | -32.274 |
| 164 | 8 | -67.213 | -32.348 |
| 165 | 8 | -66.783 | -32.511 |
| 166 | 8 | -66.546 | -32.852 |
| 167 | 8 | -66.457 | -32.556 |
| 168 | 8 | -65.315 | -32.363 |
| 169 | 8 | -66.442 | -32.096 |
| 170 | 8 | -67.035 | -31.696 |
| 171 | 8 | -67.080 | -31.236 |
| 172 | 8 | -67.124 | -30.880 |
| 173 | 8 | -67.510 | -30.376 |
| 174 | 8 | -67.777 | -30.138 |
| 175 | 8 | -66.709 | -29.501 |
| 176 | 8 | -66.724 | -28.922 |
| 177 | 8 | -66.620 | -28.655 |
| 178 | 8 | -66.397 | -28.922 |
| 179 | 8 | -66.071 | -29.634 |
| 180 | 8 | -65.211 | -31.518 |
| 181 | 8 | -64.143 | -31.547 |
| 182 | 8 | -63.669 | -31.710 |
| 183 | 8 | -63.832 | -31.577 |
| 184 | 8 | -63.965 | -31.206 |
| 185 | 8 | -63.165 | -30.761 |
| 186 | 8 | -63.150 | -30.539 |
| 187 | 8 | -63.135 | -30.302 |
| 188 | 8 | -63.016 | -30.020 |
| 189 | 8 | -63.209 | -29.723 |
| 190 | 8 | -64.025 | -30.420 |
| 191 | 8 | -64.929 | -30.435 |
| 192 | 8 | -64.470 | -30.213 |
| 193 | 8 | -65.048 | -29.649 |
| 194 | 8 | -63.565 | -29.530 |
| 195 | 8 | -63.076 | -29.530 |
| 196 | 8 | -62.764 | -29.100 |
| 197 | 8 | -62.616 | -28.567 |
| 198 | 8 | -63.995 | -28.507 |
| 199 | 8 | -64.677 | -28.285 |
| 200 | 8 | -65.389 | -28.240 |
| 201 | 8 | -64.484 | -27.647 |
| 202 | 8 | -64.811 | -27.528 |
| 203 | 8 | -63.728 | -27.603 |
| 204 | 8 | -64.484 | -26.802 |
| 205 | 8 | -63.921 | -26.105 |
| 206 | 8 | -62.838 | -26.120 |
| 207 | 8 | -62.571 | -26.639 |
| 208 | 8 | -61.296 | -27.617 |
| 209 | 8 | -61.163 | -27.410 |
| 210 | 8 | -60.169 | -27.054 |
| 211 | 8 | -60.347 | -26.965 |
| 212 | 8 | -60.406 | -26.520 |
| 213 | 8 | -59.383 | -26.223 |
| 214 | 8 | -61.326 | -26.550 |
| 215 | 8 | -60.584 | -25.897 |
| 216 | 8 | -60.733 | -25.526 |
| 217 | 8 | -61.459 | -25.215 |
| 218 | 8 | -62.735 | -24.310 |
| 219 | 8 | -63.639 | -25.586 |
| 220 | 8 | -64.054 | -25.289 |
| 221 | 8 | -64.292 | -25.171 |
| 222 | 8 | -64.069 | -24.652 |
| 223 | 8 | -64.025 | -24.385 |
| 224 | 8 | -63.965 | -24.103 |
| 225 | 8 | -63.951 | -23.762 |
| 226 | 8 | -62.067 | -23.065 |
| 227 | 8 | -59.442 | -23.213 |
| 228 | 8 | -58.612 | -22.160 |
| 229 | 8 | -59.027 | -21.908 |
| 230 | 2 | -63.491 | -24.358 |
| 231 | 2 | -67.400 | -32.450 |
| 232 | 2 | -68.217 | -32.134 |
| 233 | 2 | -68.751 | -33.304 |
| 234 | 2 | -68.066 | -33.300 |
| 235 | 2 | -64.427 | -39.363 |
| 236 | 7 | -62.462 | -21.651 |
| 237 | 4 | -62.084 | -18.472 |
| 238 | 4 | -61.248 | -20.546 |
| 239 | 6 | -60.034 | -20.489 |
| 240 | 5 | -68.796 | -37.162 |
| 241 | 5 | -68.478 | -37.480 |
| 242 | 5 | -67.835 | -30.863 |
| 243 | 5 | -66.660 | -31.837 |
| 244 | 9 | -69.916 | -40.029 |

Sources

1: Buskirk (1993)

2: Cabrera (1998)

3: The EMYSystem (<http://emys.geo.orst.edu/cgi-bin/emysmap?tn=138&cf=ijklmno>)

4: Ernst (1998)

5: Fritz et al. (2012)

6: Gonzales et al. (2006)

7: Ergueta & Morales (1996)

8: Richard (1999)

9: Waller (1986)

Table S2. Explanatory variables and model selection

| Explanatory variables included in the analysis. Each row belongs to an independent model, showing the deviance information criterion (DIC), and its difference (Δ) with the DIC of a null model (i.e. a model with only intercept parameters). Color shades are a visual help to order ΔDIC from gratest (red) to smallest (green). | | | |
| --- | --- | --- | --- |
| **Variable** |  | **DIC** | **ΔDIC** |
| Null |  | 1487.2 | 0.0 |
| bio1 | Annual Mean Temperature | 851.2 | 636.0 |
| bio2 | Mean Diurnal Range^1^ | 1253.6 | 233.6 |
| bio3 | Isothermality ^2^ | 1186.0 | 301.2 |
| bio4 | Temperature Seasonality^3^ | 1019.0 | 468.3 |
| bio5 | Max Temperature of Warmest Month | 891.5 | 595.7 |
| bio6 | Min Temperature of Coldest Month | 1163.4 | 323.8 |
| bio7 | Temperature Annual Range ^4^ | 1113.5 | 373.8 |
| bio8 | Mean Temperature of Wettest Quarter | 1002.9 | 484.3 |
| bio9 | Mean Temperature of Driest Quarter | 1385.8 | 101.4 |
| bio10 | Mean Temperature of Warmest Quarter | 945.0 | 542.2 |
| bio11 | Mean Temperature of Coldest Quarter | 988.5 | 498.7 |
| bio12 | Annual Precipitation | 1304.1 | 183.1 |
| bio13 | Precipitation of Wettest Month | 1321.9 | 165.3 |
| bio14 | Precipitation of Driest Month | 1274.6 | 212.6 |
| bio15 | Precipitation Seasonality ^5^ | 1281.8 | 205.5 |
| bio16 | Precipitation of Wettest Quarter | 1293.4 | 193.8 |
| bio17 | Precipitation of Driest Quarter | 1291.1 | 196.1 |
| bio18 | Precipitation of Warmest Quarter | 1213.4 | 273.8  235.8 |
| bio19 | Precipitation of Coldest Quarter | 1251.5 |  |
| himpact | Areas of human impact over the biosfere^6^ | 1368.6 | 118.6 |
| globedem | Altitude | 1406.2 | 81.0 |
| LAIm | Leaf Area Index | 1296.2 | 191.0 |
| iflworld | World intact forest | 1479.4 | 7.8 |
| ^1^ Mean of monthly (max temp - min temp)  ^2^ (bio2/bio7) * 100  ^3^ standard deviation *100.  ^4^ bio5-bio6  ^5^ coefficient of variation  ^6^ binomial | | | |


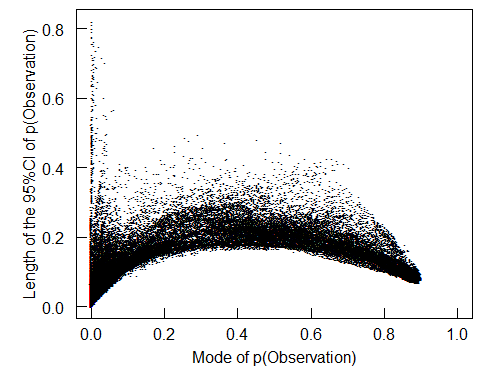


**Figure S1**: BSEL model uncertainty. Mode of probability of observation and length of the 95% CI over the study area, for the final BSEL model.

**
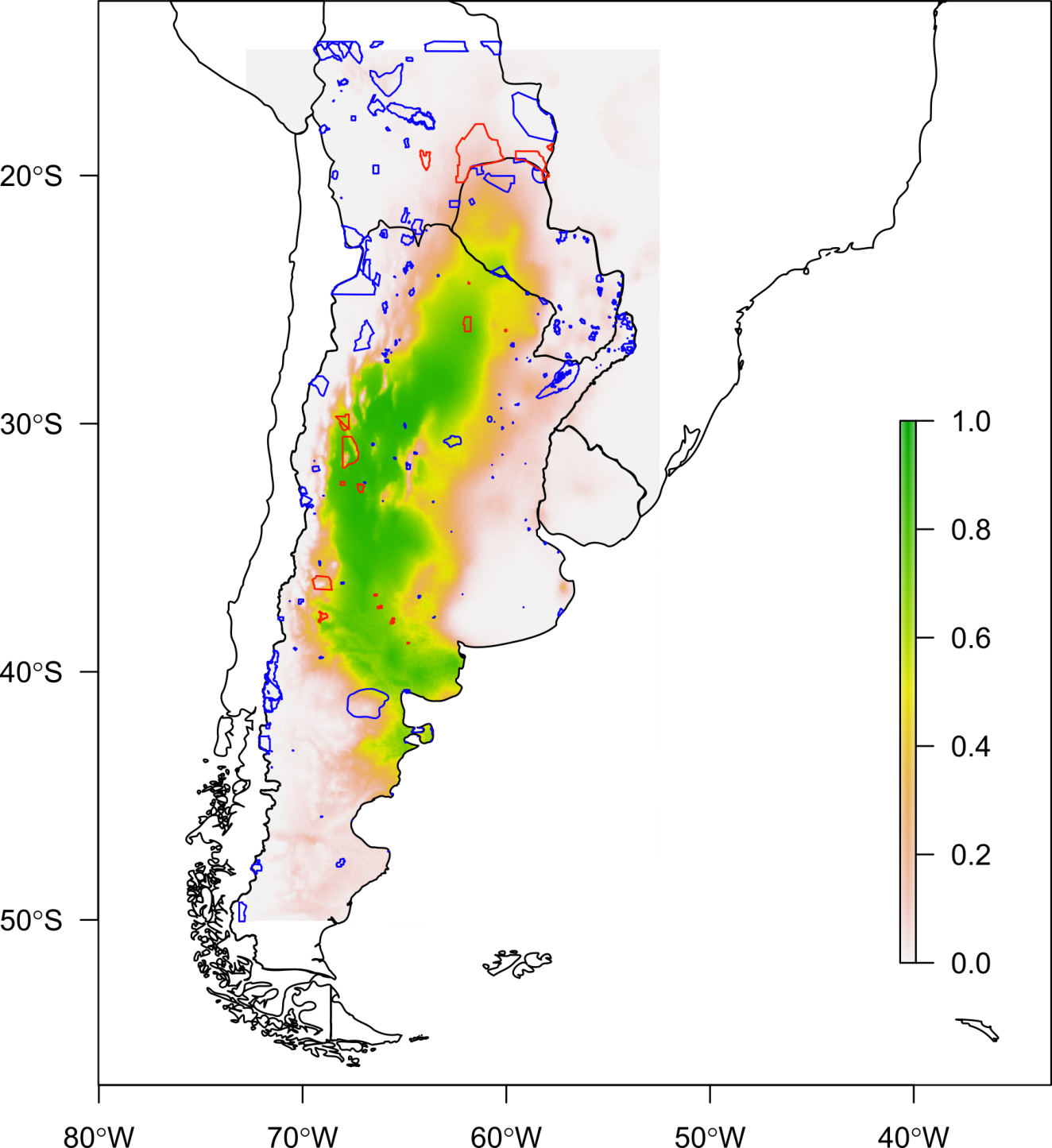
**

**Figure S2**: Predictions of the final BSEL model. The colour scale indicates the probabilities of observation. Red and blue lines show protected areas where the species has and has not been reported, respectively.

**Table S3.** Presence of *Chelonoidis chilensis* on protected areas in Argentina and Bolivia.

| **Table S3.1**: Argentinean protected areas and probabilities of observation (p) of *Chelonoidis chilensis* predicted by the Bayesian Spatially Expanded Logistic (BSEL) model. The length of the 95% credible interval is (L 95% CI) shown. | | | | | |
| --- | --- | --- | --- | --- | --- |
|  | **Protected Area** | **p(BSEL)** | **L 95% CI** | **Independent Observation** |  |
| 1 | Monte de las Barrancas | 0.89 | 0.08 | 0 |  |
| 2 | **Sierra de las Quijadas** | **0.88** | **0.11** | **1** |  |
| 3 | **Valle Fértil** | **0.87** | **0.27** | **1** |  |
| 4 | **Telteca** | **0.86** | **0.13** | **1** |  |
| 5 | Quebracho de la Legua | 0.85 | 0.09 | 0 |  |
| 6 | Chancani | 0.84 | 0.15 | 0 |  |
| 7 | **Copo** | **0.82** | **0.16** | **1** |  |
| 8 | Guasamayo | 0.74 | 0.16 | 0 |  |
| 9 | **Limay Mahuida** | **0.71** | **0.2** | **1** |  |
| 10 | **La Reforma (Univ.)** | **0.7** | **0.18** | **1** |  |
| 11 | Bahía de San Antonio | 0.7 | 0.24 | 0 |  |
| 12 | La Humada | 0.69 | 0.18 | 0 |  |
| 13 | La Reforma | 0.69 | 0.19 | 0 |  |
| 14 | **Talampaya** | **0.68** | **0.32** | **1** |  |
| 15 | **Pichi Mahuida** | **0.67** | **0.19** | **1** |  |
| 16 | Punta Delgada | 0.66 | 0.43 | 0 |  |
| 17 | **Ischigualasto (o Valle de La Luna)** | **0.65** | **0.27** | **1** |  |
| 18 | Salitral Levalle | 0.64 | 0.2 | 0 |  |
| 19 | Caleta Valdés | 0.63 | 0.22 | 0 |  |
| 20 | **Auca Mahuida** | **0.61** | **0.28** | **1** |  |
| 21 | **Lihué Calel** | **0.61** | **0.2** | **1** |  |
| 22 | Meseta de Somuncurá | 0.59 | 0.19 | 0 |  |
| 23 | **Formosa** | **0.59** | **0.18** | **1** |  |
| 24 | **El Payén** | **0.56** | **0.26** | **1** |  |
| 25 | Mar Chiquita | 0.56 | 0.19 | 0 |  |
| 26 | Santa Ana | 0.52 | 0.2 | 0 |  |
| 27 | Parque Luro | 0.45 | 0.2 | 0 |  |
| 28 | **Pampa del Indio** | **0.45** | **0.2** | **1** |  |
| 29 | Los Palmares | 0.45 | 0.18 | 0 |  |
| 30 | Laguna La Felipa | 0.43 | 0.2 | 0 |  |
| 31 | Laguna Guatrache | 0.42 | 0.23 | 0 |  |
| 32 | Agua Dulce | 0.41 | 0.24 | 0 |  |
| 33 | El Mangrullo | 0.4 | 0.32 | 0 |  |
| 34 | Vaquerías | 0.39 | 0.23 | 0 |  |
| 35 | Laguna de Llancanelo | 0.39 | 0.25 | 0 |  |
| 36 | Lagunas y Palmares | 0.37 | 0.2 | 0 |  |
| 37 | La Florida P. | 0.35 | 0.23 | 0 |  |
| 38 | Presidencia de la Plaza | 0.3 | 0.18 | 0 |  |
| 39 | Chaco | 0.29 | 0.18 | 0 |  |
| 40 | La Quebrada | 0.26 | 0.23 | 0 |  |
| 41 | Don Guillermo | 0.26 | 0.19 | 0 |  |
| 42 | La Norma | 0.25 | 0.18 | 0 |  |
| 43 | Quebrada del Portugués | 0.23 | 0.23 | 0 |  |
| 44 | El Rey | 0.22 | 0.21 | 0 |  |
| 45 | Potrero 7-B (Los Quebrachales) | 0.22 | 0.18 | 0 |  |
| 46 | Aguas Chiquitas | 0.22 | 0.21 | 0 |  |
| 47 | La Loca | 0.21 | 0.18 | 0 |  |
| 48 | Iberá | 0.2 | 0.18 | 0 |  |
| 49 | Del Medio - Los Caballos | 0.19 | 0.16 | 0 |  |
| 50 | Río Pilcomayo | 0.18 | 0.17 | 0 |  |
| 51 | Calilegua | 0.17 | 0.17 | 0 |  |
| 52 | Laguna Hu | 0.17 | 0.12 | 0 |  |
| 53 | Quebrada del Condorito | 0.14 | 0.21 | 0 |  |
| 54 | Mburucuyá | 0.12 | 0.15 | 0 |  |
| 55 | General Obligado | 0.12 | 0.12 | 0 |  |
| 56 | El Leoncito | 0.12 | 0.21 | 0 |  |
| 57 | Baritú | 0.1 | 0.23 | 0 |  |
| 58 | Campo General Belgrano | 0.1 | 0.18 | 0 |  |
| 59 | Bosques Petrificados de Jaramillo | 0.09 | 0.12 | 0 |  |
| 60 | Sierra de San Javier | 0.08 | 0.19 | 0 |  |
| 61 | Acambuco | 0.08 | 0.11 | 0 |  |
| 62 | Campo Salas | 0.08 | 0.1 | 0 |  |
| 63 | Pre Delta Diamante | 0.079 | 0.1 | 0 |  |
| 64 | Virá Pitá | 0.06 | 0.09 | 0 |  |
| 65 | Potrero de Yala | 0.06 | 0.11 | 0 |  |
| 66 | Los Cardones | 0.06 | 0.13 | 0 |  |
| 67 | Lago Urugua-í | 0.05 | 0.18 | 0 |  |
| 68 | Bosque Petrificado Sarmiento | 0.05 | 0.09 | 0 |  |
| 69 | Iguazú | 0.05 | 0.16 | 0 |  |
| 70 | Otamendi | 0.04 | 0.07 | 0 |  |
| 71 | El Tromen | 0.04 | 0.13 | 0 |  |
| 72 | Laguna Blanca (Neuquén) | 0.04 | 0.12 | 0 |  |
| 73 | Laguna Blanca | 0.04 | 0.11 | 0 |  |
| 74 | Litoral Chaqueño | 0.03 | 0.07 | 0 |  |
| 75 | Cabo Blanco | 0.03 | 0.09 | 0 |  |
| 76 | Cerro Currumahuida | 0.02 | 0.09 | 0 |  |
| 77 | Laguna Aleusco | 0.02 | 0.05 | 0 |  |
| 78 | Los Andes | 0.02 | 0.07 | 0 |  |
| 79 | Volcán Tupungato | 0.02 | 0.09 | 0 |  |
| 80 | Laguna de los Pozuelos BioRes (National) | 0.02 | 0.16 | 0 |  |
| 81 | Campo de los Alisos | 0.02 | 0.14 | 0 |  |
| 82 | Lago Puelo | 0.01 | 0.06 | 0 |  |
| 83 | Olaroz-Caucharí | 0.01 | 0.04 | 0 |  |
| 84 | Salto Encantado del Valle del Cuñá Pirú | 0.01 | 0.03 | 0 |  |
| 85 | Apipé Grande | 0.01 | 0.05 | 0 |  |
| 86 | Esperanza | 0.01 | 0.12 | 0 |  |
| 87 | Saltito | 0.01 | 0.03 | 0 |  |
| 88 | Laguna Los Juncos | 0.01 | 0.03 | 0 |  |
| 89 | Punta Lara | 0.01 | 0.03 | 0 |  |
| 90 | Nahuel Huapi Par1 | 0.01 | 0.07 | 0 |  |
| 91 | Rio Limay | 0.01 | 0.04 | 0 |  |
| 92 | Lanín | 0.01 | 0.11 | 0 |  |
| 93 | Los Alerces | 0.01 | 0.06 | 0 |  |
| 94 | Copahue-Caviahue | 0.01 | 0.17 | 0 |  |
| 95 | El Destino (P. Costero del Sur) | 0.01 | 0.03 | 0 |  |
| 96 | Urugua-í | 0.01 | 0.09 | 0 |  |
| 97 | Nahuel Huapi Parque | 0.01 | 0.04 | 0 |  |
| 98 | Nahuel Huapi Reserva | 0.01 | 0.05 | 0 |  |
| 99 | Selva Marginal de Hudson | 0.01 | 0.03 | 0 |  |
| 100 | Divisadero Largo | 0 | 0 | 0 |  |
| 101 | El Manzano Histórico | 0 | 0 | 0 |  |
| 102 | Lago Baggilt | 0 | 0 | 0 |  |
| 103 | Aconcagua | 0 | 0.03 | 0 |  |
| 104 | Cañada Molina | 0 | 0 | 0 |  |
| 105 | Nant y Fall (Arroyo Las Caídas) | 0 | 0 | 0 |  |
| 106 | Papel Misionero | 0 | 0 | 0 |  |
| 107 | Laguna Brava | 0 | 0.03 | 0 |  |
| 108 | Alto Andina de la Chinchilla | 0 | 0.03 | 0 |  |
| 109 | Colonia Benítez | 0 | 0 | 0 |  |
| 110 | Laguna La Salada | 0 | 0 | 0 |  |
| 111 | La Loma del Cristal | 0 | 0 | 0 |  |
| 112 | Moconá | 0 | 0 | 0 |  |
| 113 | Yacuy | 0 | 0 | 0 |  |
| 114 | Florencio de Basaldua | 0 | 0 | 0 |  |
| 115 | Carpincho | 0 | 0 | 0 |  |
| 116 | Los Sosa | 0 | 0 | 0 |  |
| 117 | Guaraní | 0 | 0.02 | 0 |  |
| 118 | Cruce Caballero | 0 | 0 | 0 |  |
| 119 | Cerro Azul (E.E.A.) | 0 | 0 | 0 |  |
| 120 | E.E.A. Anexo Cuartel Río Victoria | 0 | 0 | 0 |  |
| 121 | De la Sierra Crovetto | 0 | 0 | 0 |  |
| 122 | Guardaparque Horacio Foerster | 0 | 0.02 | 0 |  |
| 123 | Piñalito | 0 | 0.01 | 0 |  |
| 124 | General Belgrano | 0 | 0.01 | 0 |  |
| 125 | Los Arrayanes | 0 | 0 | 0 |  |
| 126 | Isla Botija | 0 | 0 | 0 |  |
| 127 | Sierra del Tigre | 0 | 0 | 0 |  |
| 128 | Punta Márquez | 0 | 0 | 0 |  |
| 129 | Lago Guacho | 0 | 0 | 0 |  |
| 130 | Punta Norte | 0 | 0 | 0 |  |
| 131 | Cerro Alcazar | 0 | 0 | 0 |  |
| 132 | La Florida R. | 0 | 0 | 0 |  |
| 133 | El Estero | 0 | 0 | 0 |  |
| 134 | Isla Laguna Alsina | 0 | 0 | 0 |  |
| 135 | Yabotí | 0 | 0.02 | 0 |  |
| 136 | Golfo San José | 0 | 0 | 0 |  |
| 137 | Perito Moreno | 0 | 0.01 | 0 |  |
| 138 | Los Glaciares | 0 | 0.03 | 0 |  |
| 139 | Ira Hiti | 0 | 0 | 0 |  |
| 140 | Cabo dos Bahías | 0 | 0 | 0 |  |
| 141 | Cayastá | 0 | 0 | 0 |  |
| 142 | El Pozo | 0 | 0 | 0 |  |
| 143 | Punta Pirámides | 0 | 0 | 0 |  |
| 144 | Punta Loma | 0 | 0 | 0 |  |

| **Table S3.2**: Bolivian protected areas and probabilities of observation (p) of *Chelonoidis chilensis* predicted by the Bayesian Spatially Expanded Logistic (BSEL) model. The length of the 95% credible interval is (L 95% CI) shown. | | | | | |
| --- | --- | --- | --- | --- | --- |
|  | **Protected Area** | **p(BSEL)** | **L 95% CI** | **Independent Observation** |  |
| 1 | Area de proteccion del Quebracho Colorado | 0.28 | 0.27 | 0 |  |
| 2 | **Kaa-iya del Gran Chaco** | **0.16** | **0.23** | **1** |  |
| 3 | Isiboro Securé | 0.09 | 0.8 | 0 |  |
| 4 | Tariquía | 0.09 | 0.14 | 0 |  |
| 5 | Madidi | 0.05 | 0.35 | 0 |  |
| 6 | Area de proteccion del Pino del Cerro | 0.04 | 0.13 | 0 |  |
| 7 | **Otuquis** | **0.04** | **0.08** | **1** |  |
| 8 | Pilón Lajas | 0.04 | 0.35 | 0 |  |
| 9 | Carrasco | 0.03 | 0.3 | 0 |  |
| 10 | Cordillera de Sama | 0.03 | 0.19 | 0 |  |
| 11 | **Iñao** | **0.03** | **0.04** | **1** |  |
| 12 | Apolobamba | 0.02 | 0.28 | 0 |  |
| 13 | Cotapata | 0.02 | 0.12 | 0 |  |
| 14 | Cotapata | 0.02 | 0.12 | 0 |  |
| 15 | Amboró | 0.01 | 0.04 | 0 |  |
| 16 | Amboró | 0.01 | 0.05 | 0 |  |
| 17 | El Palmar | 0.01 | 0.02 | 0 |  |
| 18 | Estancias San Rafael | 0.01 | 0.03 | 0 |  |
| 19 | Noel Kempff Mercado | 0.01 | 0.02 | 0 |  |
| 20 | Ríos Blanco y Negro | 0.01 | 0.03 | 0 |  |
| 21 | San Matías | 0.01 | 0.01 | 0 |  |
| 22 | Toro Toro | 0.01 | 0.02 | 0 |  |
| 23 | Tunari | 0.01 | 0.05 | 0 |  |
| 24 | Cavernas del Repechón | 0 | 0 | 0 |  |
| 25 | Cerro Tapilla | 0 | 0 | 0 |  |
| 26 | Eduardo Avaroa | 0 | 0.02 | 0 |  |
| 27 | Estación Biológica del Beni | 0 | 0 | 0 |  |
| 28 | Flavio Machicado Viscarra | 0 | 0 | 0 |  |
| 29 | Huancaroma | 0 | 0 | 0 |  |
| 30 | Incacasani Altamachi | 0 | 0.04 | 0 |  |
| 31 | Las Barrancas | 0 | 0 | 0 |  |
| 32 | Llica | 0 | 0 | 0 |  |
| 33 | Madidi | 0 | 0 | 0 |  |
| 34 | Mallasa | 0 | 0 | 0 |  |
| 35 | Mirikiri | 0 | 0 | 0 |  |
| 36 | Sajama | 0 | 0.01 | 0 |  |
| 37 | Tuni Condoriri | 0 | 0.01 | 0 |  |
| 38 | Yura | 0 | 0.01 | 0 |  |
